# Do the Changes in the Serum Levels of IL-2, IL-4, TNF*α*, and IL-6 Reflect the Inflammatory Activity in the Patients with Post-ERCP Pancreatitis?

**DOI:** 10.1155/2008/481560

**Published:** 2008-07-21

**Authors:** Guldem Kilciler, Ugur Musabak, Sait Bagci, Zeki Yesilova, Ahmet Tuzun, Ahmet Uygun, Mustafa Gulsen, Sema Oren, Cagatay Oktenli, Necmettin Karaeren

**Affiliations:** ^1^Department of Gastroenterology, Gulhane Military Medical Academy, School of Medicine, Etlik, 06010 Ankara, Turkey; ^2^Department of Immunology, Gulhane Military Medical Academy, School of Medicine, Etlik, 06010 Ankara, Turkey; ^3^Department of Internal Medicine, Gulhane Military Medical Academy, Haydarpasa Training Hospital, Uskudar, 34668 Istanbul, Turkey

## Abstract

*Background*. Acute pancreatitis is the major complication of endoscopic retrograde cholangiopancreatography (ERCP) procedure and there are some reports showing cytokine changes in ERCP-induced pancreatits. *Goals*. To investigate the association between early changes (within 24 hours) in the serum interleukin (IL)-2, IL-4, tumor necrosis factor (TNF)*α*, and IL-6 levels and the development of post-ERCP pancreatitis. *Study*. Forty five consecutive patients who underwent therapeutic ERCP and 10 patients with acute pancreatitis without ERCP were enrolled to the study. Serum concentrations of IL-2, IL-4, TNF*α*, and IL-6 were determined immediately before, 12 hours and 24 hours after ERCP. *Results*. Seven of the 45 patients (15.5%) developed post-ERCP pancreatitis. The levels of IL-4 at 24 hours after ERCP were significantly lower in the patients with post-ERCP pancreatitis than in those without pancreatitis, while TNF*α* levels at 12 hours after ERCP were higher in the complicated group than those of the uncomplicated group. The ratios of TNF*α*/IL-4 at 12 and 24 hours after ERCP were found significantly higher in the patients with post-ERCP pancreatitis than in those without pancreatitis. IL-6 in the complicated patients was found significantly increased at 24 hours after ERCP. *Conclusions*. The enhancement of serum TNF*α* and IL-6 levels in the patients with ERCP-induced pancreatitis reflects the inflammatory activity. Additionally, these cytokines together with IL-4 can be used in clinical laboratory monitoring of ERCP.

## 1. INTRODUCTION

Acute pancreatitis is an important and critical complication of endoscopic retrograde cholangiopancreatography (ERCP) [1]. The incidence of post-ERCP pancreatitis has been reported between 1% and 10% in various publications [2]. ERCP is mostly performed on an outpatient base. Therefore, early diagnosis of post-ERCP pancreatitis is important for hospital admission and management. However, recognition of post-ERCP pancreatitis based on clinical datas is not reliable [3]. Several biochemical and immunologic markers were determined as predictor of pancreatic inflammation after ERCP [4]. These markers are serum/urine amylase, trypsinogen, trypsinogen activation peptide, C-reactive protein (CRP), and some cytokines [2, 4].

The studies show that the serum levels of tumor necrosis factor *α* (TNF*α*), interleukin (IL)-1*β*, IL-6, and IL-8 are significantly increased in acute pancreatitis [2, 4, 5]. Pancreatic injury is mediated by the release of these proinflammatory cytokines. After trypsinogen activation into trypsin, a local inflammation is initiated which results in the local production of inflammatory mediators [5]. Interestingly, serum levels of anti-inflammatory molecules such as IL-10, IL-1*β* receptor antagonist, and soluble IL-2 receptor (sIL-2r) were also found significantly higher in patients with acute pancreatitis [4, 5]. These findings support that these opposite effective cytokines play roles together in the pathogenesis of acute pancreatitis.

A dynamic balance exists between proinflammatory (pro-) and anti-inflammatory cytokines. The timing of cytokine release, the local milieu, the presence of antagonistic or synergistic factors, and the cytokine receptor density determines the net effect of any cytokine [6]. Polarization of T helper (Th) lymphocytes into functional Th1 and Th2 subsets is one of the main factors that determine the direction of the balance between pro- and anti-inflammatory cytokines [7, 8]. While Th1 type cells produce high levels of inflammatory cytokines including IL-2, TNF*α*, and interferon*γ* (IFN*γ*), Th2 type cells produce anti-inflammatory cytokines including IL-4, IL-5, IL-6, IL-10, and IL-13 [9]. Both type cells also produce lesser amounts of TNF*α*, granulocyte-macrophage colonystimulating factor (GM-CSF), and IL-3 [9]. Th1-type cytokines are essential for the cell-mediated immune responses against intracellular microorganisms [10]. However, Th2-type cytokines participate in the development of humoral immunity against extracellular microorganisms [10]. These 2-type cytokines have cross-regulatory activity [9, 10]. IL-4 and IL-10 lead to the inhibition of Th1-type immune responses by down-regulating the production of macrophage-derived IL-12. Whereas, IFN*γ* alteres the balance of Th1-/Th2-type immune responses in favour of Th1 by suppressing the Th2-type immune responses. It was also known that the imbalance in the Th1/Th2 cytokine immune response is related to the pathogenesis of some chronic inflammatory diseases such as rheumatoid arthritis (RA), a Th1-type disease, and systemic lupus erythematosus (SLE), a Th2-type disease [11].

The aim of this study was to investigate the association between early changes (within 24 hours) in the serum IL-2, IL-4, TNF*α*, and IL-6 concentrations and occurence of subsequent pancreatitis complication after ERCP.

## 2. MATERIALS AND METHODS

Forty five consecutive patients (20 men and 25 women; mean age: 54.8 years; SD:15.9) who underwent therapeutic ERCP were enrolled to the study. Exclusion and inclusion criteria of this study are detailed in Table 1. Control group consisted of 10 patients with acute pancreatitis without ERCP (7 men and 3 women; mean age: 48.7 years; SD:17.3). Etiological factors in the control group were gallstones in 4, drug useage in 3, and alcohol in 2 patients. A certain etiological factor could not be determined in 1 patient.

All the patients who underwent ERCP had normal serum levels of CRP and amylase, before ERCP procedure. Serum concentrations of IL-2, IL-4, TNF*α*, and IL-6 were determined immediately before 12 hours and 24 hours after ERCP. ERCP was performed by the same operator after premedication. Olympus JF-240 electronic duodenoscope (Olympus Optical Co., Ltd, Tokyo, Japan) was used for ERCP. Radiologic images were taken by using C-arm x-ray device (Philips, BV 300, Philips Medical Systems, Best, The Netherlands). Antibiotic prophylaxis with Cephazolin Sodium was started for all patients undergoing ERCP. After an overnight fast, all patients were sedated with 10 mg i.v. dormicum. For cytokine measurements, serum samples from each patient were stored at −70°C until run. However serum amylase and CRP levels were determined on the day of sampling. Serum amylase levels were determined by enzymatic color test and upper limit of normal range was accepted as 90 L/U according to our laboratory standarts. Serum CRP levels were measured using a nephelometer (Behring BN II Analyzer, Dade Behring, Marburg, Germany) and the high-sensitivity reagent kit. A level <6 mg/L for CRP was accepted as normal. Serum levels of IL-2, IL-4, TNF*α*, and IL-6 were determined using ELISA kits (Bender MedSystems GmbH, Vienna, Austria). Interassay and intra-assay coefficients of variation for each assay were, respectively, as follows: 8 and 5.2% for IL-2; 5.6 and 4.8% for IL-4; 7.4 and 6.9% for TNF*α*; and 5.2 and 3.4% for IL-6.

The diagnostic criteria for post-ERCP pancreatitis were epigastric pain during 24 hours after ERCP, at least three-fold increase in upper limit of normal level of serum amylase and/or acute pancreatitis seen in the abdominal ultrasonography and computed tomography [12]. All patients were informed about the aim and procedures of the study and gave their consent. The study was approved by the Ethical Committee of Gulhane Military School of Medicine.

All statistical analyses were performed using SPSS (SPSS 11.5, SPSS Inc., Chicago, IL, USA) statistical package. For the tests of normality, we used Kolmogorov-Smirnov test. Results are expressed as median (range). The Friedman test was used for multiple statistical comparisons. The Wilcoxon test was used as a post hoc test if the Friedman test is statistically significant. Mann-Whitney U test was used to compare the mean or median values of cytokines, CRP, and amylase in blood in the various study groups. To investigate the relations among the variables, we used Pearson or Spearman's rank correlation test, which was appropriate. A *P* value < .05 was considered to be statistically significant.

## 3. RESULTS

Seven of the 45 patients (15.5%) developed post-ERCP pancreatitis. Two of them had severe pancreatitis and 5 patients had mild disease according to Atlanta criteria [13]. First symptoms were occured a mean of 5.2 hours after ERCP in these 7 patients.

Serum amylase levels at 12 and 24 hours after ERCP were significantly higher in the patients with post-ERCP pancreatitis than in those without pancreatitis (Table 2). The levels of amylase were also significantly higher in the control group with acute pancreatitis than in those of the levels before and after ERCP in the patients without post-ERCP pancreatitis. While serum amylase levels before and 24 hours after ERCP were significantly lower in the patients with post-ERCP pancreatitis than those of the control group with acute pancreatitis, the amylase levels at 12 hours after ERCP in the patients with post-ERCP pancreatitis were not different from those of the control group with acute pancreatitis. CRP levels before and within 24 hours after ERCP in the patients who underwent ERCP and those of the control group with acute pancreatitis were not different from one other. In serial measurements of complicated and uncomplicated patients, serum amylase levels at 12 and 24 hours after ERCP were higher than those of the basal levels (*P* = .018 and *P* < .001, resp.), and the levels of amylase at 24 hours after ERCP were also higher than those of the levels at 12 hours after ERCP (*P* = .018 and *P* < .001, resp.). There was no significant difference among the CRP levels in the serial measurements of complicated and uncomplicated patients.

**Figure 1:**
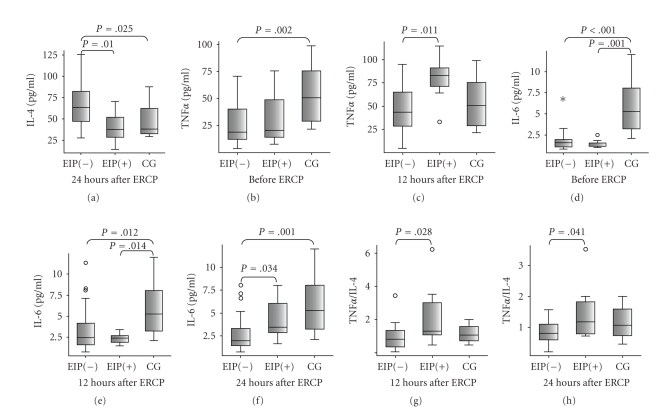
The levels of IL-4 (a) and TNF (b), (c), IL-6 (d), (e), (f) and the ratios of TNF/IL-4 (g), (h) in the patients with ERCP-induced pancreatitis (EIP+), patients without ERCP induced pancreatitis (EIP−), and the control group (CG) with acute pancreatitis without ERCP. Boxes show the ranges of 1st and 3rd quartiles and extreme values. Horizontal bars represent median values. The differences between two groups were evaluated by Mann-Whitney U test. *P* values were indicated above the boxes when a level of significance less than or equal to .05 was reached in comparisons of study groups.

Significant differences in IL-4, IL-6 and TNF*α* levels at 12 and 24 hours after ERCP were found among the patients who underwent ERCP and the control group patients. The levels of IL-4 at 24 hours after ERCP in the patients with post-ERCP pancreatitis and the control group patients were significantly lower than in those without ERCP-induced pancreatitis (Table 2, Figure 1(a)). While TNF*α* levels in the control group patients were higher than those of the levels before ERCP in the patients with and without post-ERCP pancreatitis (Table 2, Figure 1(b)), the levels of TNF*α* at 12 hours after ERCP in the patients with and without post-ERCP pancreatitis were not different from the control patients (Table 2, Figure 1(c)). However those TNF*α* levels in the complicated group were higher than those of the levels at 12 hours after ERCP in the uncompicated group (Table 2, Figure 1(c)). There was no other significant difference among complicated and uncomplicated patients before and within 24 hours after ERCP and control group patients with respect to serum IL-4 and TNF*α* concentrations. Addionally, IL-2 levels before and within 24 hours after ERCP in the complicated and uncomplicated patients and the control patients were not different from one another. While the levels of IL-6 were higher in the control group patients than those of the levels before and 12 hours after ERCP in both patients group who underwet ERCP (Table 2, Figures 1(d), 1(e)), IL-6 levels at 24 hours after ERCP in the patients with post-ERCP pancreatitis were not different from the control patients but higher than those of the patients without post-ERCP pancreatits (Table 2, Figure 1(f)). On the other hand, the ratios of TNF*α*/IL-4 at 12 and 24 hours after ERCP were found significantly higher in the complicated patients than those of the uncomplicated patients but not diffrent from the control patients (Table 2, Figures 1(g), 1(h)). There was not any difference with respect to IL-2/IL-4 ratio among all patient groups.

**Figure 2:**
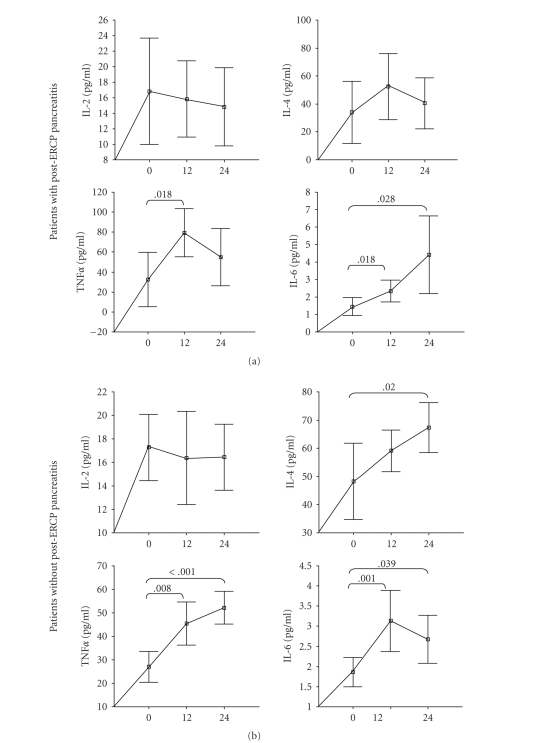
Serial concentrations of IL-2, IL-4, TNF and IL-6 before and within 24 hours after ERCP in the patients (a) with post-ERCP pancreatits and (b) without pancreatitis. Bars represent mean ± 95% CI. The differences between two groups were evaluated by Mann-Whitney U test. *P* values were indicated above the boxes when a level of significance less than or equal to .05 was reached in comparisons of study groups.

Statistical significant differences were observed among the levels of IL-4, TNF*α*, and IL-6 in serial measurements before and within 24 hours after ERCP in the patients who underwent ERCP (Table 2, Figure 2). In the patients with post-ERCP pancreatitis and without pancreatitis, significantly higher levels of TNF*α* and IL-6 were found at 12 hours after ERCP compared to the levels before ERCP. In the patients without post-ERCP pancreatitis, the levels of IL-4, TNF*α*, and IL-6 at 24 hours after ERCP were also higher than those of the basal levels. However, in the complicated group, only IL-6 levels at 24 hours after ERCP were higher than the basal levels.

In the complicated patients, basal IL-6 levels positively correlated with basal CRP levels, but negatively correlated with basal IL-4 levels (*r* = 0.812; *P* = .048, *r* = 0.847; *P* = .016, resp.). In the complicated patients, IL-4 and CRP levels at 24 hours after ERCP were also negatively correlated with each other (*r* = 0.900; *P* = .037). In the patients without post-ERCP pancreatitis, a positive correlation was found between IL-4 and TNF*α* levels at 24 hours after ERCP (*r* = 0.397; *P* = .018), but there was a negative correlation between IL-4 and IL-6 levels at 12 hours after ERCP (*r* = 0.392; *P* = .018).

## 4. DISCUSSION

Acute pancreatitis is the most common serious complication of ERCP [1]. The incidence of post-ERCP pancreatitis was found 15.5% in our study. This incidence is comparable to that (2 to 15%) of other reports [14, 15].

In our study, serum amylase levels at 12 and 24 hours after ERCP were significantly higher in the patients with post-ERCP pancreatitis than in those without pancreatitis. Additionally, the levels of amylase were also significantly higher in the control group with acute pancreatitis than in those of the levels before and 24 hours after ERCP and those of the levels before and within 24 hours after ERCP in the patients with post-ERCP pancreatitis and without pancreatitis, respectively, but not different from those of the levels 12 hours after ERCP in the complicated group. Hyperamylasemia associated with abdominal pain has been evaluated as a reliable indicator of post-ERCP pancreatitis [16, 17]. However, serum amylase levels are commonly elevated in uncomplicated ERCPs. Therefore, it seems that serum amylase measurement alone is not a well indicator in predicting the development of pancreatitis after ERCP. In our study, increased amylase levels at 12 and 24 hours after ERCP were found in serial measurements of patients with post-ERCP pancreatitis and without pancreatitis compared to the basal levels. The levels of amylase at 24 hours after ERCP in both patient groups were also higher than those of the levels at 12 hours after ERCP. The serial changes of amylase levels in patients without pancreatitis suggest the existance of subclinical pancreatic damage. It is well known that serum amylase levels rise in reaction to manipulations during ERCP in the majority of patients [4].

C-reactive protein (CRP) is one of the acute-phase reactant that increases during systemic inflammation. We did not find any significant difference in the levels of CRP before and after ERCP in comparison between patients with post-ERCP pancreatitis and without pancreatitis or among serial measurements of complicated and uncomplicated patients. CRP levels in both ERCP groups were also not different from the control group patients. Our findings may be explained by CRP being a late marker in the laboratory monitoring of post-ERCP pancreatitis [18]. Its changes are commonly initiated at least 24 hours after ERCP [4]. However, we did not investigate the changes of CRP levels in the late phase after ERCP. Therefore, our results showed that CRP does not increase in the early phase after ERCP.

There are several speculative conclusions related to cytokine investigations in ERCP in the literature. Our study provides a new insight in ERCP-induced pancreatitis with regard to results. We found that the levels of TNF*α* were increased at 12 hours after ERCP in the patients with post-ERCP pancreatitis compared with those of the patients without pancreatitis. There was no significant difference in TNF*α* concentrations before and 24 hours after ERCP between complicated and uncomplicated patients. In Chen et al.'s study, TNF*α* was found significantly increased at 8 and 24 hours after ERCP in the patients with post-ERCP pancreatitis [2]. However, Messmann et al. did not find significant difference in the TNF*α* measurements of patients with post-ERCP pancreatitis [19].

We found that the serum levels of IL-4 at 24 hours after ERCP were lower in the patients with post-ERCP pancreatitis and control group patients than in those of the patients without post-ERCP pancreatitis. It is well known that IL-4 plays a crucial role in Th2-cell development [9, 10]. Additionally, IL-4 has potent anti-inflammatory properties and inhibits secretion of IL-1*β* and TNF*α* by monocytes. It may be said that low level of IL-4 in the patients with post-ERCP panceratitis and control patients causes the relative or absolute dominance of inflammatory state. We investigated the serum levels of inflammatory cytokines including IL-2, TNF*α*, IL-6 and IL-2/IL-4, and TNF*α*/IL-4 ratios before and after ERCP in order to identify the dominance of inflammatory or anti-inflammatory state at different time points of ERCP. According to our results, neither IL-2 levels nor IL-2/IL-4 ratios were different from one another among the patients who underwent ERCP and the control patients. Although there is not any report on IL-2 levels in before and after ERCP, increased sIL-2r levels was observed in a study in the patients with acute panceratitis [20]. We did not also find any report investigating the relation between IL-4 and ERCP in the literature. However, in Chen et al.'s study, the other anti-inflammatory cytokine IL-10 was reported as a marker with respect to reflect the severity of acute pancreatitis [21]. This study seems contrary to our study with regard to indicating the dominance of an anti-inflammatory state after ERCP.

In our study, the evidences suggested TNF*α* dominance in the patients with post-ERCP pancreatitis were high levels of TNF*α* at 12 hours after ERCP and high ratio of TNF*α*/IL-4 at 12 and 24 hours after ERCP. The levels of TNF*α* at 12 hours after ERCP in the complicated patients were not different from the control patients. These findings are compatible with the studies reported by Chen et al. [2] and Devière et al. [22] but contrary to Oezcueruemez-Porsch et al.'s study [23]. In the first study, the levels of TNF*α* were reported to be significantly increased at 8 and 24 hours after ERCP in the patients with post-ERCP pancreatitis [2]. The increased TNF*α* levels in patients with post-ERCP pancreatitis were also observed in Devière et. al.'s study. However, TNF*α* levels at any time of ERCP were not detectable in the patients with evidence of ERCP-induced pancreas damage. Although some controversial results in the literature, our findings together with Chen et. al.'s study suggest that there was a bias towards increase of TNF*α* within 24 hours after ERCP.

In our study, IL-6 levels at 24 hours after ERCP in the patients with post-ERCP pancreatitis were not different from the control group patients but higher than those of the patients without post-ERCP pancreatits. However IL-6 levels before and 12 hours after ERCP in both patients with ERCP-induced pancreatitis and without pancreatitis were not observed differently from each other. As compatible with our results, in many studies, IL-6 has been reported as a good predictor for the development of pancreatitis after ERCP [2, 4, 23, 24].

We observed that the serial changes occur in IL-4, TNF*α*, and IL-6 after ERCP. According to our findings, TNF*α* and IL-6 levels in the complicated patients were found to be incerased at 12 hours after ERCP compared to those of the basal levels. In the complicated patients, IL-6 levels at 24 hours after ERCP were also higher than those of the basal levels. Besides the increased levels of TNF*α* and IL-6 at 12 and 24 hours after ERCP were found in our uncomplicated patients compared to those of the basal levels, the levels of IL-4 were also found increased at 24 hours after ERCP than those of the basal levels. Our results related to the serial measurements of the cytokines support the existance of inflammatory activity and subclinical pancreatic damage in patients without ERCP-induced pancreatitis. Our findings with respect to serial IL-6 changes are compatible with the studies reported by Chen et al. [2] and Messmann et al. [19]. In their studies, maximal concentration of IL-6 in the ERCP procedure was found 24 hours after ERCP.

We observed reasonable correlations among the measured parameters in the patients with or without ERCP-induced pancreatitis. CRP was negatively correlated with IL-4 levels at 24 hours after ERCP in the patients with post-ERCP pancreatitis that supports the dominance of inflammatory activity at this time after ERCP. Surprisingly, basal IL-6 levels in the patients with post-ERCP pancreatitis positively correlated with basal CRP levels, but negatively correlated with basal IL-4 levels. On the other hand, IL-4 positively correlated with TNF*α* at 24 hours after ERCP in the uncomplicated patients, but negatively correlated with IL-6 at 12 hours after ERCP. These findings indirectly support the existance of systemic immune activation in the patients without ERCP-induced pancreatitis. Diffrerent results related to the correlations among the cytokines, pancreatic enzymes, and CRP levels in the ERCP procedure were reported in the literature. In the Chen et al.'s study, serum IL-6 was found significantly correlated with serum amylase at 8 and 24 hours after ERCP [2]. In another study, serum matrix metalloproteinase 9 (MMP9) level in severe acute pancreatitis was positively correlated with TNF*α* and CRP levels [25].

## 5. CONCLUSIONS

In conclusion, the enhancement of serum TNF*α* and IL-6 levels in the patients with ERCP-induced pancreatitis reflects the inflammatory activity. Additionally, IL-4, IL-6, and TNF*α* can be used in clinical laboratory monitoring of ERCP. However, it should be considered that the Th1 or Th2 cytokine dominance before and after ERCP is one of the main factors determining the balance between pro- and anti-inflammatory statuses of organism. Some conflicting cytokine results about ERCP-induced pancreatitis in the literature may depend on the direction of Th polarization at the sampling time for serum cytokine levels. Therefore, the other Th cytokines (IFN*γ*, IL-12, IL-18, IL-5, IL-13) may influence the inflammatory activity after ERCP, and can be a topic of the future study. In addition, it seems that the presenting the cytokine profile is important to effective usage of anti-inflammatory cytokines and cytokine inhibitors in ERCP-induced pancreatitis. On the other hand, monitoring interval in the post-ERCP period should be longer than 24 hours.

## Figures and Tables

**Table 1:** Exclusion and inclusion criteria of the patients.

Exclusion criteria	Inclusion criteria
Emergency ERCP	Choledocholithiasis
Previous therapeutic ERCP	Pancreato-biliary malignancy
Previous surgery of the upper GI tract	Cholestasis of unknown origin
Acute pancreatitis at time of study	Cholangitis
Known history of chronic pancreatitis	Tumor of papilla
Less than 18 years of age	Others
Known history of mental disease	
Unable to give informed consent	
Female who is pregnant	
Underlying chronic liver disease	
Abnormal coagulation tests (PTT < 75% or INR > 1.5 or APTT > 35)	
Immunocompromized patients	

**Table 2:** Comparisons of IL-2, IL-4, TNF*α*, IL-6, CRP and amylase levels and IL-2/IL-4 and TNF*α*/IL-4 ratios in the patients who underwent ERCP and control group with acute pancreatitis without ERCP.

	Patients with post-ERCP pancreatitis (*n* = 7) **(1)**			Patients without pancreatitis (*n* = 38) **(0)**			Patients with acute pancreatitis without ERCP (*n* = 10) **(2)**
Time (hour)	**0.**	**12.**	**24.**	**0.**	**12.**	**24.**	
IL-2 (pg/mL)	15.6 (19.9)	14.3 (14.1)	12.7 (15.5)	15.1 (43.9)	12.7 (76.0)	15.1 (46.0)	11.4 (13.0)
IL-4 (pg/mL)	29.0 (69.1)	63.6 (67.5)	**37.7 (56.4)** ^(a)^	37.7 (146.8)	55.9 (83.8)	**63.6 (98.0)** ^(b)^	**37.9 (58.4)** ^(c)^
TNF*α* (pg/mL)	20.2 (67.8)	**82.9 (81.9)** ^(f)^	44.9 (77.5)	**18.6 (66.9)** ^(d)^	**43.6 (90.5)** ^(g)^	51.8 (72.1)	**50.3 (77.5)** ^(e)^
IL-6 (pg/mL)	**1.2 (1.4)** ^(h)^	**2.4 (1.9)** ^(k)^	**3.4 (6.4)** ^(m)^	**1.6 (5.8)** ^(j)^	**2.4 (10.5)** ^(l)^	**1.9 (7.3)** ^(n)^	**5.2 (9.8)** ^(i)^
IL-2/IL-4	0.62 (2.03)	0.33 (2.30)	0.40 (0.90)	0.44 (26.81)	0.24 (2.61)	0.23 (0.71)	0.3 (0.61)
TNF*α*/IL-4	0.56 (10.0)	**1.31 (5.77)** ^(o)^	**1.18 (2.80)** ^(r)^	0.67 (91.83)	**0.82 (3.38)** ^(p)^	**0.81 (1.35)** ^(s)^	1.0 (1.53)
CRP (mg/dL)	10.2 (49)	17.0 (33)	10 (47)	3.9 (212)	8.0 (395)	6.7 (342)	12.0 (42)
Amylase (U/mL)	**50 (74)** ^(t)^	**1114 (1806)** ^(x)^	**460 (552)** ^(z)^	**47 (179)** ^(v)^	**131 (673)** ^(y)^	**79 (290)** ^(zz)^	**1357 (2558)** ^(u)^

*P* values: .010 for (a) versus (b); .025 for (b) versus (c); .002 for (d) versus (e); .011 for (f) versus (g); .001 for (h) versus (i); <.001 for (j) versus (i); .014 for (k) versus (i); .012 for (l) versus (i); .034 for (m) versus (n); .001 for (n) versus (i); .028 for (o) versus (p); .041 for (r) versus (s); .001 for (t) versus (u); <.001 for (v) versus (u); <.001 for (x) versus (y); <.001 for (y) versus (u); <.001 for (z) versus (zz); <.001 for (zz) versus (u); .001 for (z) versus (u).
